# Improved Loss Function for Mass Segmentation in Mammography Images Using Density and Mass Size

**DOI:** 10.3390/jimaging10010020

**Published:** 2024-01-09

**Authors:** Parvaneh Aliniya, Mircea Nicolescu, Monica Nicolescu, George Bebis

**Affiliations:** Computer Science and Engineering Department, College of Engineering, University of Nevada, Reno, 89557 NV, USA; monica@unr.edu (M.N.); bebis@cse.unr.edu (G.B.)

**Keywords:** breast cancer, mass segmentation, loss function, density, adaptive sample-level prioritizing loss, hybrid loss

## Abstract

Mass segmentation is one of the fundamental tasks used when identifying breast cancer due to the comprehensive information it provides, including the location, size, and border of the masses. Despite significant improvement in the performance of the task, certain properties of the data, such as pixel class imbalance and the diverse appearance and sizes of masses, remain challenging. Recently, there has been a surge in articles proposing to address pixel class imbalance through the formulation of the loss function. While demonstrating an enhancement in performance, they mostly fail to address the problem comprehensively. In this paper, we propose a new perspective on the calculation of the loss that enables the binary segmentation loss to incorporate the sample-level information and region-level losses in a hybrid loss setting. We propose two variations of the loss to include mass size and density in the loss calculation. Also, we introduce a single loss variant using the idea of utilizing mass size and density to enhance focal loss. We tested the proposed method on benchmark datasets: CBIS-DDSM and INbreast. Our approach outperformed the baseline and state-of-the-art methods on both datasets.

## 1. Introduction

Breast cancer is one the most common cancer types among females [1], and has been one of the main contributors to the high mortality statistics of cancer in females. The early detection of cancer has a significant impact on the improvement of the five-year survival rate compared with late-stage cancer [2]. Thanks to the cost-effectiveness and availability of mammography as a screening tool, it is one of the main tools for the early identification and treatment of the disease.

In recent years, the ability of deep-learning-based methods in automated feature extraction has enhanced the performance of automated breast cancer identification, despite limitations such as data scarcity in this domain. However, reading the mammography for findings has specific challenges, such as misdiagnosis or high cost of second readers. Therefore, using computer-aided diagnosis (CAD) systems could be highly beneficial to radiologists and patients.

Initially, CAD systems were designed based on traditional machine learning approaches [3,4,5,6,7]; however, the excellent performance of deep-learning-based approaches [8,9,10,11] in recent years has encouraged the research community in the medical domain to utilize and customize them to the special needs in this domain [12]. Despite the reported effectiveness of using deep learning methods for the identification of breast cancer [13,14,15], there are several challenges, including pixel class imbalance and various breast densities constraining the performance of the methods. Addressing these problems could greatly enhance the performance of the methods. There are various abnormalities in the mammography images, including masses, asymmetrical breast tissue, micro-calcification, and architectural distortion of breast tissue [16]. Among the abnormality types, masses are reportedly major contributors to breast cancer [16]. Therefore, in this paper, we aim to address the identification of masses that generally has two forms: mass detection [17,18,19,20] and mass segmentation [21,22,23,24,25]. Mass segmentation provides more comprehensive information, including border information; hence, in this paper, the main task is mass segmentation.

Generally, deep-learning-based methods are the current state-of-the-art in most computer vision tasks. Among the various factors affecting the performance of deep-learning methods, the architecture of the network and loss function are of vital importance. A majority of the proposed methods aim to improve and customize the architecture of the network [21,22,23,24,25,26], which has proven to be effective. Another line of research attracting major attention from the research community is the customization of the loss function. As the loss function presents the objective of the network, adjustments to the definition of the loss could improve the training [27,28,29,30,31]. Loss functions for segmentation, in general, compare the pixels in the prediction and ground truth masks. While some loss functions consider pixels as individual entities (pixel-level losses) [30,32], others consider the neighboring pixels when calculating the loss for a central pixel (region-level losses) [33,34]. This, in turn, allows the loss function to incorporate the dependencies between the pixels in the loss calculation. Pixel-level losses mostly differ in the way they treat the TP, TN, FP, and FN rates. A common trend when using the proposed losses for mass segmentation is to use hybrid losses, which constitute a weighted sum of two or more losses.

While they are effective, there are several aspects for improvement in the currently available losses. Firstly, they neglect to use the extra information available in the dataset in the training to improve the performance. Secondly, the definition of the loss function is static; for example, the weights for the losses in the hybrid setting are fixed, despite the fact that the severity of a problem, such as the pixel class imbalance or the density, can vary across samples.

In this paper, we propose novel loss functions, taking the aforementioned shortcomings into consideration to improve the performance of the method in the presence of pixel class imbalance and higher densities. To this end, we propose two loss categories that use extra information in the data to address the problems in different ways. While they utilize different strategies, one aspect they share is being sample-level, meaning that the formulation of the losses depends on the information in each sample. In addition, both approaches use extra information to prioritize the more suitable loss term for each sample in the hybrid loss. Therefore, the general name for these losses is “Adaptive Sample-Level Prioritizing” (ASP) losses.

The first loss function, which utilizes the ratio of the mass in each sample for re-weighting the loss term in a hybrid setting is referred to as R-ASP loss (R stands for the ratio), as it uses the ratio of the mass size to the image size for prioritizing the loss terms over each other. The second loss, named D-ASP (D stands for density), is inspired by the same idea, but differs with respect to the information it uses for the re-weighting and the losses incorporated into the hybrid loss. Both losses have been tested on benchmark datasets, the Curated Breast Imaging Subset of DDSM (CBIS-DDSM) and INbreast, using AU-Net as the baseline architecture. In addition, we propose customizing the ASP method for the selection of the focusing parameter in focal loss to examine the potential of ASP for a single loss. The results of the experiments demonstrate a robust improvement in the performance of the method to varying degrees across the proposed ASP losses, outperforming the state-of-the-art methods.

The contributions of this paper are as follows:

- Proposing a general framework for adaptive sample-level prioritizing losses.
- Introducing two variations of ASP loss that alleviate the limitation of performance caused by pixel class imbalance and density categories.
- Customizing focal loss to use the ratio and density for the selection of the focusing parameter.
- Evaluating the proposed losses on CBIS-DDSM [35] and INbreast [16] datasets using AU-Net architecture.
- Performing ablation study on INbreast to investigate the impact of different parameters.
- Comparing the ASP losses with traditional hybrid loss and state-of-the-art methods.

In the following sections, a comprehensive review of the related work is provided, followed by the proposed method and a detailed explanation of each of the losses. Next, the experimental settings for testing the proposed losses, as well as an analysis of the results, is provided. Finally, the paper ends with Section 5 and Section 6.

## 2. Related Work

When it comes to the outstanding performance gained by the adoption of deep learning as an end-to-end approach for automated feature extraction and segmentation, mass segmentation is no exception. As a result of the transformation in recent years, the majority of the proposed methods fall into the deep-learning category, which is the main target of this paper. Therefore, in this section, mass segmentation approaches [36,37,38,39,40,41,42] using deep-learning-based methods are first reviewed. In addition, as the main contribution of the paper is to propose new ways to incorporate additional information in the hybrid loss setting for binary segmentation, reviewing the losses for this task is also essential; hence, the second subsection is devoted to reviewing the binary segmentation losses, mostly focusing on the ones proposed for medical applications.

### 2.1. Review of Mass Segmentation Approaches

Supervised deep-learning-based mass segmentation methods could be performed on the region of interest (ROI) [43,44] of the masses or the full field of view (FOV) (or entire mammography image). As the main task in this paper was to perform segmentation on the image, only the previous work for mass segmentation on the whole view mammography images was reviewed in this section. This was because the proposed losses were for alleviating problems such as the pixel class imbalance that was presented in the whole-view mammography image.

While the authors of [45,46] were among the very first to design a segmentation method using deep learning, U-Net [12] is a pioneering work in the realm of deep-learning-based segmentation for medical applications that is still inspiring new methods to this day.

The fully convolutional network (FCN) [45] introduced a network with skip connections and end-to-end training for segmentation tasks. The skip connections solved the problem of losing more precise location information by combining the location information “where” from the encoding part with the semantic information “what” from the decoder through the summation of corresponding pooling and up-sampling layers in two paths. U-Net extended the core idea of FCN by proposing a symmetric encoder–decoder network that differed from FCN in the sense that the skip connections were more present throughout a network with a symmetric structure in the encoder and decoder parts. In addition, instead of summation, U-Net utilized concatenation for the feature maps and further processed the resulting feature maps.

Following the success of U-Net and FCN, in recent years, research [41,47,48,49,50,51,52,53,54,55] in the medical imaging domain has exceeded the performance limits of segmentation through the adaptation and advancement of these approaches. For instance, Drozdzal et al. [56] explored the idea of creating a deeper FCN by adding a short skip connection to the decoder and encoder paths. Zhou et al. [49] developed more sophisticated skip connections to create features that were more semantically compatible before merging the feature maps from the contacting and expanding paths. Another architecture named UNet++ [57] introduced the idea of using an ensemble of U-Nets with varying depths to address the problem of unknown optimal depth of variations of U-Net and also proposed a new skip connection that enabled the aggregation of features from previous layers as opposed to the traditional way of combining features from the same semantic level.

Hai et al. [58] moved beyond general modification to the U-Net architecture and improved the design while considering the challenging features of the mammography data, such as the diversity of shapes and sizes. To this end, they utilized an Atrous Spatial Pyramid Pooling (ASPP) module in the transition between the encoder and decoder paths. The ASPP block consisted of 1×1 conv plus three atrous convolutions [59] with sample rates of 6, 12, and 18; the outputs for these layers were concatenated and fed into a 1×1 conv. FC-DenseNet [60] was selected as the backbone of the method, which is an adaptation of Excellent DenseNet [61] for the segmentation task by constructing a U-Net shape network. Shuyi et al. [62] is another U-Net-based approach based on the idea of utilizing densely connected blocks for mass segmentation. In the encoder, the path is constructed from densely connected CNNs [61]. In the decoder, gated attention [52] modules are used when combining high and low-level features, allowing the model to focus more on the target. Another line of research within the scope of multi-scale studies is [63], in which the generator is an improved version of U-Net. Multi-scale segmentation results were created for three critics with identical structures and different scales in the discriminator. Ravitha et al. [25] developed an approach to use the error of the outputs of intermediate layers (in both encoder and decoder paths) relative to the ground truth labels as a supervision signal to boost the model’s performance. In every stage of the encoder and decoder, attention blocks with upsampling were applied to the outputs of the block. The resulting features were linearly combined with the output of the decoder and incorporated into the objective criterion of the network to enhance the robustness of the method.

Sun et al. [21] introduced an asymmetric encoder–decoder network (AU-Net). While in the encoder path, res blocks (three conv layers with a residual connection) were used; in the decode path, basic blocks (including two conv layers) were utilized. The main contribution of the paper was a new upsampling method. In the new attention upsampling (AU) block, high-level features were upsampled through dense and bilinear upsampling. Then, the low-level was combined with the output of dense upsampling through element-wise summation. The resulting feature maps from the previous step were concatenated with the output of bilinear upsampling and were fed into a channel-wise attention module. Finally, the input of the channel-wise attention module was combined with its output by channel-wise multiplication. To address the relatively low performance of U-Net on small-size masses, Xu et al. [22] proposed using a selective receptive field module with two modules—one for generating several receptive fields with different sizes (MRFM) and one for selecting the appropriate size of the receptive field (MSSM) in the down-sampling path of the network. MRFM, in separate paths, applies 3×3 and 5×5 conv on the input image. MSSM takes the two sets of feature maps from the MRFM module, and after combining the features from multi-scale paths in the selection module, the input is processed in three branches with global average pooling and two local context branches using convolutions with different kernel sizes. AU-Net [21] was the baseline method in this study and ARF-Net [22] was used for the comparison.

### 2.2. Loss Functions for Mass Segmentation

The loss function is one of the essential components in the design of a successful deep-learning approach. For the mass segmentation task, which is designed as pixel-level labeling of the input image, the loss function compares the labels in the ground truth and predicted masks for each pixel to penalize the mismatches. With regard to the mass segmentation task, masses could have various sizes (from less than one percent of the image to considerably larger sizes), shapes, and appearances. One of the greatest challenges for the mass segmentation task is the pixel class imbalance problem. In addition, one extra factor that is specific to mammography is that mammography images have various tissue density categories, and the identification of masses becomes harder as the category number increases. Therefore, in this section, we focus on the losses proposed for binary segmentation for medical images and, specifically, those useful for the aforementioned challenges. Considering the paper’s contribution, we categorized the losses into pixel-level and region-level losses. In this paper, we refer to the loss functions that measure the similarity by only comparing a pair of pixels as pixel-level losses. On the contrary, the second group of losses that incorporate regional information, such as the similarity of the surrounding pixels in the calculation of the loss for a pair of pixels, are referred to as region-level losses.

#### 2.2.1. Pixel-Level Losses

One of the widely used losses in this category is binary cross entropy (BCE) [32], which penalizes mismatch between pixels. As the pixel level computation in BCE makes it vulnerable to bias toward correctly predicting the majority class in the presence of pixel class imbalance, which is specifically common in medical applications, several attempts have been made to ensure the BCE loss is robust to the problem [64]. In this category, focal loss [65] aims to diminish the effect of pixel class imbalance by reducing the impact of easy samples. Additional variants of focal loss can be found in [28,66]. Balanced cross entropy [67,68] controls the impact of classes by using the ratio of pixels in each class to the total as balancing coefficients in BCE terms. Dice loss [69] is robust to the pixel class imbalance problem [70], as it measures correctly classified pixels within true and predicted positive classes. Dice loss is also one of the common losses used for segmentation, and there are several variants of dice loss [29,71,72]. Tversky loss [30] balances the contribution of the false positive and the false negative terms by assigning coefficients to each of the false positive and false negative terms.

#### 2.2.2. Region-Level Losses

In this category, which was initially proposed for image quality assessment, the Structural Similarity Index Measure (SSIM) [73] was utilized in segmentation loss for medical image segmentation [55]. SSIM measures the similarity of images in pixel and region levels. Regional Mutual Information (RMI) [33] and Structural Similarity Loss (SSL) [34] are two examples of region-level losses developed specifically for segmentation. The definition of the region can be refined, as in [74,75], where each region is represented by a cell in a grid, or it is dynamically adjusted, for example through sliding window approaches, to calculate the loss for each of the pixels in the ground truth. It should be noted that both of these losses consider a fixed-size window around each pixel as the region and belong to the latter category.

Inspired by the influence of the structural term in SSIM, the authors of SSL [34] introduced a method for capturing structural similarity by weighting the cross-entropy of every two pixels based on the structural error (error between two regions, which indicates the degree of linear correlation), while ignoring easy pixels and emphasizing pixels with a high error by thresholding the error rate. In the same category, RMI [33] considered a region around a centering pixel as a multi-dimensional point (for a 3×3 region, it will be a 9D point) and then maximized the MI between multidimensional points.

Using two or several losses in a hybrid setting is a common practice in the literature. For instance, all three region-level losses have been tested in a hybrid setting in combination with other losses. In this category, several losses [27,30,31,76] have been proposed to combine beneficial losses for a certain task. As the weighted sum of BCE and dice, combo loss [27] was proposed to control the contribution of false positive and false negative rates by a weighting strategy within the BCE loss term.

## 3. Materials and Methods

**Figure 1:**
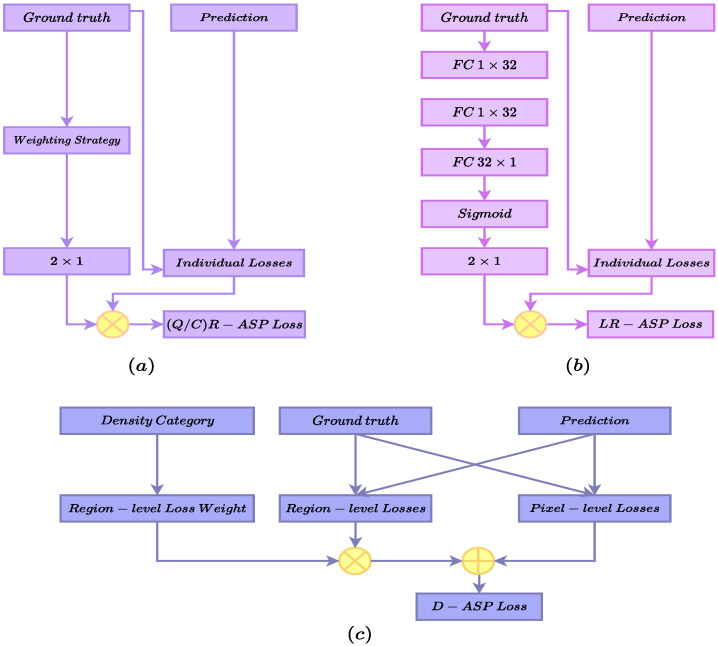
An overview of the proposed ASP losses. (**a**) presents the QR-ASP and CR-ASP losses. (**b**) shows the LR-ASP loss and the sub network for learning the weights. (**c**) presents the D-ASP loss calculation module.

BCE is a widely used loss function for binary segmentation. When applied to mass segmentation in medical imaging, and mammography images specifically, the presence of pixel class imbalance, which is a domain-specific property of the data, can lead to bias. This bias is prone to favoring correctly classifying the major class, as it considers the contribution of both classes as equal while measuring the similarity of the ground-truth and the prediction masks. Therefore, a common practice is to use it in a hybrid setting alongside other losses that will help to mitigate this problem. For example, dice loss ignores the true negative in the calculation, which makes it immune to favoring the majority class, but opens the door for other issues, such as instability in training. Therefore, combining these losses is beneficial with respect to both losses. In recent years, there has been a surge in hybrid losses for mass segmentation and medical imaging in general due to the proven performance gain they provide. However, they mostly use static weights for the losses and only utilize the information in the ground truth and predicted masks. We argue that hybrid loss could benefit from adaptive weighting, which can be customized for each sample. In addition, we propose using the available information, such as the mass ratio and ACR density, for calculating the loss as a re-weighting signal for the adaptive sample-level prioritizing losses. ACR stands for American College of Radiology (ACR), which developed a standard categorization for density for mammography images containing four categories, from low to high densities. In the following sections, we delve into the details of using mass ratio and ACR density in the proposed loss functions (shown in Figure 1).

### 3.1. Ratio as Weighting Signal in the Adaptive Sample-Level Prioritizing Loss

One of the challenges in mass segmentation is that masses can occupy anywhere from less than 1% of the image to a significantly larger area. This means the severity of the pixel class imbalance will vary greatly between the samples. Therefore, the ratio of the mass to the image size can be considered a direct indicator of the severity of the pixel class imbalance problem for individual samples. We propose using this ratio as a sample-level signal to adaptively re-weight losses. One crucial aspect is how to incorporate the adaptive signal into the loss. We propose utilizing the relationship between losses and the severity of the pixel class imbalance. For instance, BCE is not suitable for small ratios in which the pixel class imbalance is high. On the other hand, dice loss can capture the differences between pixels better for samples with low ratios, in which the pixel class imbalance is high. Therefore, based on this observation, we formulate the Ratio-ASP (R-ASP) loss in a way that BCE has an inverse relationship with the mass ratio of the sample, and the dice has a direct relationship with the ratio, as presented in Equation (1).

(1) $$L_{R\text{-}ASP}^{i}=\left(I_{Dice}+\left(1-p^{i}\right)\gamma \right)L_{Dice}^{i}+\left(I_{BCE}+\left(p^{i}\right)\gamma \right)L_{BCE}^{i}$$

Here, $L_{R\text{-}ASP}^{i}$ is the R-ASP loss for the *i*th sample. $I_{Dice}$ and $I_{BCE}$, are initial weights for dice and BCE, respectively. $L_{Dice}$ and $L_{BCE}$ are dice and BCE losses, respectively. *p* indicates whether the *i*th sample belongs to the large sample group, and γ is the hypermarapeter controlling the effect of the ratio in R-ASP. It should be noted that using the raw ratio will make the training unstable, as weighting will be sensitive to even a very small amount of change. Hence, we propose grouping the samples into two categories, small and large, based on the ratio of the mass. In this regard, the dividing value and strategy becomes important. In this section, we introduce three different ways to divide the samples into two categories (calculating $p^{i}$).

#### 3.1.1. Quantile-Based R-ASP Loss

An intuitive way to divide the samples according to the ratios is to divide them according to the quantile to which they belong based on the value of the median, as shown in Equation (2), which we name Quantile-based R-ASP (QR-ASP).

(2) $$p_{Q}^{i}=\{\begin{array}{cc} 0 & r^{i}\in Q\_1\\ 1 & \mathrm{Otherwise} \end{array}$$

Here, $r^{i}$ is the ratio of the *i*th sample and $Q_{1}$ is the first quantile. In the ablation study section, we also tested this variation with the mean value of the ratios for all of the samples as a dividing factor; for future reference, we used the term Value-based ASP (VR-ASP) for these experiments.

#### 3.1.2. Cluster-Based R-ASP Loss

The distribution of the data is skewed for both of the datasets; hence, we speculated that QR-ASP might not have been the best solution in such scenarios, which were sampled from real-world data. Therefore, in this variation, we proposed dividing the samples based on the cluster to which each sample belonged, as in Equation (3), which we termed Cluster-based R-ASP (CR-ASP). Figure 1a presents the QR/CR-ASP variants.

(3) $$p_{C}^{i}=\{\begin{array}{cc} 0 & r^{i}\in C_{s}\\ 1 & \mathrm{Otherwise} \end{array}$$

Here, $C_{s}$ is the center of the cluster for the small ratio group. The advantage of CR-ASP was that, in this case, the division of the samples was a better representative of the distribution of data in the case of skewed data compared with using the QR-ASP method.

#### 3.1.3. Learning-Based R-ASP Loss

Finally, in the last version of R-ASP, we proposed learning the weights for the hybrid loss. To this end, a parameterized subnetwork was used who used the inputs of ground truth, and its outputs were the weights for the BCE and dice losses. The subnetwork is presented in Figure 1b and the loss is presented in Equation (4), which we named learning-based R-ASP (LR-ASP). We speculate that learning the weights during the training might provide the method with an automated way to capture the differences between samples.

(4) $$L_{LR\text{-}ASP}^{i}=f{\left(y,w\right)}_{1}L_{Dice}^{i}+f{\left(y,w\right)}_{2}L_{BCE}^{i}$$

Here $f{\left(y,w\right)}_{1}$ and $f{\left(y,w\right)}_{2}$ are the outputs of the module that learn the relation between the samples and loss terms, respectively. *w* presents the weights of the LR-ASP module.

### 3.2. ACR Density as Weighting Signal in the Hybrid Adaptive Sample-Level Loss

While the ratio of mass assists the loss in handling the class imbalance problem, there is another problem specific to mammography images that makes the segmentation task challenging, which is the ACR density category of the breast. In samples with more dense tissues, the identification of masses is more demanding, and, in some cases, normal tissues might hide the masses. In most cases, the proposed methods and losses do not take ACR density into consideration for mass segmentation, while it is available for each examination (using the ACR or other methods for labeling the density). In this section, we proposed a new hybrid loss function that included the region-level and pixel-level losses. The pixel-level losses were sufficient for less dense samples, while region-level losses were more appropriate for samples with higher-density categories. This was because considering the neighboring pixels in loss calculation helped with better segmentation for more dense samples, in which the segmentation was more challenging. In order to signal the loss to prioritize one of the loss terms according to the density category of the sample, we proposed using ACR density as an adaptive signal for the hybrid loss function (including pixel-level and region-level losses).

#### 3.2.1. Pixel-Level Loss Term

As mentioned before, we referred to losses that considered the pixels as independent entities as pixel-level losses. From this category, we used a weighted sum of BCE and dice loss, which is a commonly used hybrid pixel-level loss. The pixel-level loss was prioritized more for the samples that had a lower ACR density and provided the benefit of both BCE (Equation (5)) and dice (Equation (6)) losses to some extent. The pixel-level term is presented in Equation (7).

(5) $$L_{BCE}=-\left(ylog\left(\hat{y}\right)+\left(1-y\right)log\left(1-\hat{y}\right)\right)$$

(6) $$L_{Dice}=1-\frac{\sum_{j=1}^{H\times W}{\hat{y}}_{j}y_{j}+\epsilon }{\sum_{j=1}^{H\times W}{\hat{y}}_{j}+\sum_{j=1}^{H\times W}y_{j}+\epsilon }$$

(7) $$L_{HP}=\alpha L_{Dice}+\beta L_{BCE}$$

Here, *y* and $\hat{y}$ are the ground truth and the predicted segmentation, respectively. α and β are the weighting parameters in the hybrid loss denoted as $L_{HP}$ in Equation (7). In our experiments, both α and β were set to one. *W* and *H* are the width and height of the images, respectively.

#### 3.2.2. Region-Level Loss Term

Region-level loss in this paper refers to the category of losses that incorporate the surrounding pixels in the calculation of the loss for a centering pixel. We selected SSIM and RMI losses that, based on our experiments, performed well in a combined setting. It should be noted that ACR density only had a connection with the region-level losses; therefore, it was only used for prioritizing the region-level term. One of the region-level losses was RMI in Equation (8), which measured the similarity of multi-dimensional points generated by comparing a centering pixel and its surrounding pixels (in the ground truth and predicted masks), which allowed the method to achieve consistency between the predicted and true mask beyond what using only the pixel-level comparison could measure.

(8) $$L_{RMI}\left(Y_{m};\hat{Y_{m}}\right)=\int_{S}\int_{\hat{S}}f\left(y,\hat{y}\right)\log\left(\frac{f(y,\hat{y})}{f\left(y\right)f\left(\hat{y}\right)}\right)\;dy\;d\hat{y}$$

Here, $Y_{m}$ and $\hat{Y_{m}}$ are the multi-dimensional points that are generated from a window with a fixed size around a centering pixel capturing the dependency of the neighboring pixels. The subscript *m* refers to the ‘multi-dimensional’ term. *S* and $\hat{S}$ are the support sets for the ground truth and prediction masks, respectively. f(y) and $f(\hat{y})$ represent the probability density functions of the ground truth and prediction masks, respectively. $f(y,\hat{y})$ computes the joint PDF. The implementation details of the RMI loss are available in [33] and the code is publicly available.

The second region-level loss used in this paper was SSIM-based loss, as shown in Equation (9).

(9) $$L_{SSIM}\left(Y_{p};{\hat{Y}}_{p}\right)=1-\frac{\left(2\mu_{y_{p}}\mu_{{\hat{y}}_{p}}+C_{1}\right)\left(2\sigma_{y\hat{y}}+C_{2}\right)}{\left(\mu_{y_{p}}^{2}\mu_{{\hat{y}}_{p}}^{2}+C_{1}\right)\left(\sigma_{y}^{2}+\sigma_{\hat{y}}^{2}+C_{2}\right)}$$

Here, $Y_{p}$ and $\hat{Y_{p}}$ represent patches in the ground truth and prediction masks, respectively, in which subscript *p* refers to the ‘patch’ term. μ and σ are the mean and variance for the corresponding patches, respectively. $\sigma_{y\hat{y}}$ is the covariance of the two patches. More details (including the selection of $C_{1}$ and $C_{2}$) are available in the original paper [73]. Finally, the hybrid region-level loss is presented in Equation (10).

(10) $$L_{HR}=\eta L_{RMI}+\tau L_{SSIM}$$

In the hybrid region-level loss $L_{HR}$ (Equation (10)), $L_{RMI}$ and $L_{SSIM}$ are the RMI and SSIM losses, respectively. The hyperparameters η and τ represent the weighting coefficients. Figure 1c and Equation (11) present the density-based ASP (D-ASP) loss.

(11) $$L_{D\text{-}ASP}^{i}=d^{i}\theta^{T}L_{HR}^{i}+L_{HP}^{i}$$

$L_{D\text{-}ASP}^{i}$ is the final D-ASP loss for the *i*th sample. θ is the prioritizing vector of the weights assigned to each density category, and $d^{i}$ denotes a one-hot encoding of the density category of the *i*th sample. $d^{i}\theta^{T}$ will re-weight the region-level loss term, which determines the importance of the region-level term according to the density category of the *i*th sample.

### 3.3. Adaptive Sample-Level Focal Loss

Up to this point, we have investigated the idea of using size and ACR density as the re-weighting signal in hybrid losses. In this section, we aim to explore the possibility of using the ratio category as a sample-level value to control the impact of pixels in the samples in a single loss. To this end, we selected focal loss, which was appropriate for this purpose as it was based on BCE and was developed for the same purpose. In the focal loss presented in Equation (12), υ is the focusing parameter, which reduces the impact of loss for easy-to-classify pixels. Assuming that the segmentation of the larger masses was easier as they are more salient compared with the smaller masses, we proposed increasing υ for the large category and decreased it for the small category. This enabled the loss to adaptively change the hyper-parameter that was considered to be fixed for all samples in the in the original focal loss.

(12) $$L_{F}\left({\hat{Y}}_{t}\right)=-{\left(1-{\hat{Y}}_{t}\right)}^{\upsilon }\cdot \log\left({\hat{Y}}_{t}\right)$$

Here, υ is the focusing parameter and ${\hat{Y}}_{t}$ presents the model’s estimated probability for the mass. In the modified focal loss, there will be two values for focusing parameters, corresponding to the large and small groups instead of a fixed value, as presented in Equation (13). This, in turn, allows for the sample-level adaptation of focal loss according to the property of the sample.

(13) $$\upsilon_{ASP}=\{\begin{array}{cc} \upsilon_{c_{1}} & r^{i}\in C_{1}\\ \upsilon_{c_{2}} & r^{i}\in C_{2} \end{array}$$

Here, $c_{1}$ and $c_{2}$ are the two categories for the samples and $r^{i}$ is the ratio of the *i*th sample. We used the name Single (loss) R-ASP (SR-ASP) loss. $\upsilon_{ASP}$ presents the adaptive υ. The same idea could be used for ACR density, as the density of the sample was related to the hardness of the segmentation. This variant was called Single (loss) D-ASP (SD-ASP). It should be noted that the ratio and density were not directly connected to each pixel, but to each image. Therefore, the ASP version of the focal loss indicated that the effect of an easy pixel in an easy sample should be lower and vice versa.

### 3.4. Evaluation Metrics

Dice Similarity Coefficient, (DSC, Equation (14)), Relative Area Difference, (ΔA, Equation (15)), Sensitivity (Equation (16)), and Accuracy (Equation (17)) were selected as the evaluation metrics in all of the experiments due to the complementary information they provided. As masses occupied a trivial portion of the images, using accuracy solely would not be an informative mean to evaluate the method. Hence, using an additional metric such as sensitivity that allowed us to measure the false negative rate, which is important in medical applications, is vital. DSC measures the ratio of the correctly predicted positive pixels over the number of positive areas in both the ground truth and the prediction mask, considering the false positive rate in the calculations alongside false negatives. Therefore, we also included DSC in the evaluation metrics. Finally, the relative area difference, which measures the difference in the sizes of the actual and predicted masses compared to the size of true mass (smaller value for ΔA, indicates closer sizes for masses and is better), was considered in the experiments. In all of the tables, the best results are highlighted in bold font. In the figures for the examples of the segmentation results, the green and blue borders present the ground truth and the prediction of the methods, respectively.

(14) $$DSC=\frac{2TP}{2TP+FP+FN}$$

(15) $$\Delta A=\frac{\left|(TP+FP)-(TP+FN)\right|}{TP+FN}$$

(16) $$Sensetivity=\frac{TP}{TP+FN}$$

(17) $$Accuracy=\frac{TP+TN}{TP+TN+FN+FP}$$

Here, TP, TN, FP, and FN represent true positive, true negative, false positive, and false negative rates, respectively.

### 3.5. Datasets and Experimental Setting

INbreast and CBIS-DDSM were used in this paper to test the proposed method. Normalization and resizing to 256×256 were performed on both datasets without extra data augmentation or image enhancement. For the baseline approach, the batch size was set to 4; the learning rate was set to 0.0001 in all experiments. For INbreast, from the 410 images (for 115 cases), 107 images containing masses were used in this study. As the dataset was small, following a common practice for using INbreast, we used a 5-fold cross-validation. The dataset was randomly divided into training (80%), validation (10%), and test (10%) sets. CBIS-DDSM had a total of 1944 cases, in which 1591 images contained masses that were utilized in our experiments. We used the official train (1231) and test (360) split of the dataset with 10% of the training data as a validation set, which was randomly selected. The CBIS-DDSM images had some artifacts; therefore, the artifacts were removed in the preprocessing step.

### 3.6. Comparison of Dataset Characteristics

**Figure 2:**
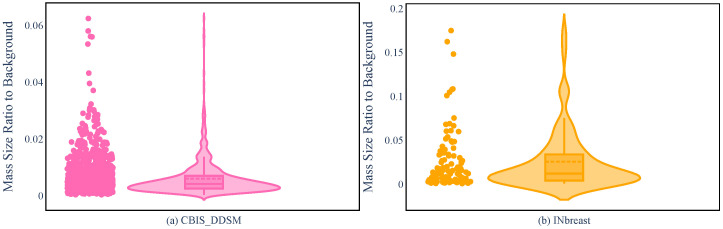
Violin plots for the CBIS-DDSM (**a**) and INbreast (**b**) based on the mass ratio.

**Figure 3:**
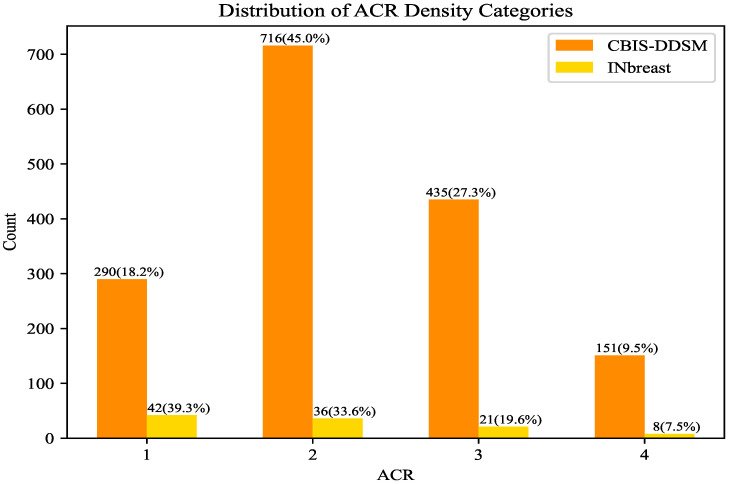
Comparison of INbreast and CBIS-DDSM based on the distribution of the density categories. ACR density increases according to the category number.

INbreast and CBIS-DDSM are different in many respects, ranging from the dataset size to the accuracy of the segmentation. However, regarding the mass size that was used in R-ASP, it was important to analyze the differences in the distribution of the sizes in the two datasets. To this end, the distributions of mass ratios in the datasets are depicted in Figure 2. CBIS-DDSM (Figure 2a) has a smaller range compared with the INbreast dataset (Figure 2b). The smaller length of the interquartile for the CBIS-DDSM dataset indicated less diversity in the ratio for the majority of the masses. In addition, a closer mean and median in the CBIS-DDSM dataset could be a sign of less skewed data for ratio. When comparing the performance of R-ASP, considering these differences could be helpful; regarding D-ASP, the difference in the distribution of the density categories is relevant information that is provided in Figure 3. Generally, for both of the datasets, most of the samples had lower densities.

## 4. Experimental Results

### 4.1. Ablation Study

**Figure 4:**
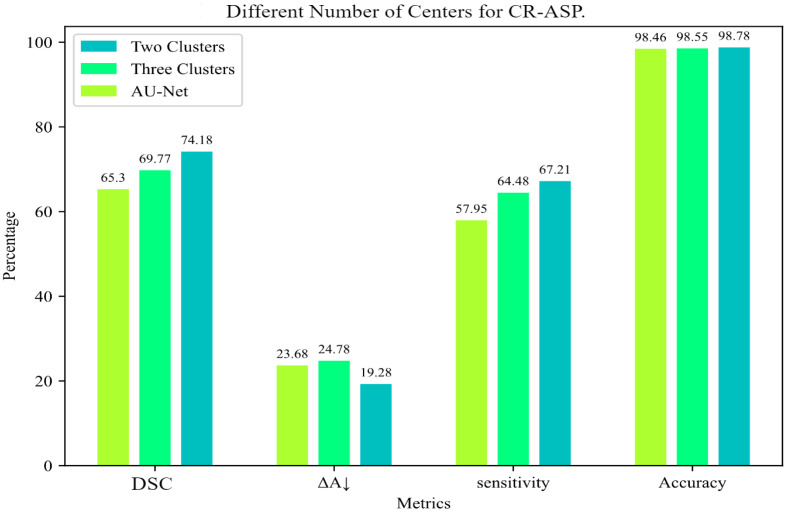
Comparison of different numbers of clusters for CR-ASP loss for INbreast dataset.

For CR-ASP, the number of centers was a hyperparameter. We tested CR-ASP with two and three clusters to select the right value. As shown in Figure 4, using two clusters showed a better performance in comparison with three clusters.

Regarding VR-ASP, SR-ASP, and SD-ASP, we conducted experiments on the INbreast dataset, and the results are presented in Table 1. As shown in the table, VR-ASP, with a mean value of 0.025, provided a modest improvement in the baseline method. Regarding VR-ASP, in which the mean value of the ratio was utilized for grouping the samples while improving on the baseline method (shown by the name hybrid in Table 1), VR-ASP achieved the lowest improvement in DSC and accuracy in the R-ASP category, but the sensitivity and ΔA were better than both LR-ASP and QR-ASP. Regarding SD-ASP and SR-ASP, we conducted an experiment to identify the best υ value for the focal loss, which was 0.5 for our experiments. SR-ASP and SD-ASP provided a considerable boost in the performance of the focal loss in a single loss setting, as shown in Table 1. SR-ASP surpassed focal loss in all the metrics and performed better than SD-ASP in terms of DSC and accuracy. It should be noted that while DSC was better for SR-ASP compared with SD-ASP, the sensitivities were comparable. This might indicate a better reduction in false negative rate for the SD-ASP, which was aligned with the experimental results in the following sections.

For the SR-ASP, the value of υ was set to 0.25 for samples belonging to the small group, and 0.5 for the large group. For SD-ASP, the values were [0.2, 0.25, 0.3, 0.35] for categories from 1 to 4, respectively. For VR-ASP, the values for γ in Equation (2) were 0.25 and 0.25 and the $I_{Dice}$ and $I_{BCE}$ were considered one.

### 4.2. Comparison with State-of-the-Art Method

In order to examine the strengths and weaknesses of the proposed R-ASP and D-ASP losses, they were tested on INbreast and CBIS-DDSM datasets. AU-Net and ARF-Net were selected for comparison. AU-Net was the baseline method; thus, it was essential to compare the performance of ASP variations with it. As AU-Net used the hybrid loss function, including dice and BCE, comparison with AU-Net provided information about the impact of ASP losses. ARF-Net is another method in which different scales were incorporated into the design of the network; this was related to our study as it incorporated the idea of designing the network in a way that leveraged different sizes. For AU-Net, the official implementation was publicly available, and the training setting was closely followed by the description in the paper. We implemented ARF-Net to the best of our understanding of the ARF-Net paper. For the region-level term in D-ASP, publicly available implementations of RMI and SSIM were utilized. No pre-training or data augmentation were used in any of the experiments. For R-ASP the hyperparameters $I_{Dice}$ and $I_{BCE}$ were set to 0.125 and 0.25 for INbreast and CBIS-DDSM, respectively. For γ, a value between [0.25, 0.35] was used with different values for the dice and BCE terms. For the cluster-based strategy, the number of centers was a hyperparameter, for which 2 was selected. Regarding D-ASP, the coefficients were θ=[0.5,0.5,0.85,0.95] and θ=[0.25,0.25,0.85,0.95] for the INbreast and CSIB-DDSM, respectively. η, τ, β were set to 1; α was set to 2 and 2.5 for INbreast and CBIS-DDSM, respectively. All of the hyperparameters were selected through experimental evaluation.

#### 4.2.1. Experimental Results for R-ASP

Table 2 and Table 3 presented the results of the experiments for R-ASP using the INbreast and CBIS-DDSM datasets, respectively. For the INbreast dataset, for the best-performing variation, CR-ASP, the improvements were as follows: DSC: +8.86%, ΔA: −4.4%, Sensitivity: +9.26%, and Accuracy: +0.32%. Considering Figure 2, due to the distribution of the sizes in the dataset, as expected, the CR-ASP version outperformed QR-ASP. While QR-ASP improved on the baseline generally, we speculate that its subpar performance compared with the two other R-ASP variations could be due to the fact that the groupings of the samples to small and large categories using QR-ASP were not suitable considering the distribution of the sizes in the dataset. Regarding LR-ASP, while it did not boost the performance to the same level as CR-ASP, it surpassed AU-Net, ARF-Net, and QR-ASP in all of the metrics. The achieved level of performance for LR-ASP compared with the baseline indicated that the learned weights for the loss terms were useful for prioritizing the loss terms. In general, CR-ASP seemed to provide a better connection between the samples and the loss terms.

**Figure 5:**
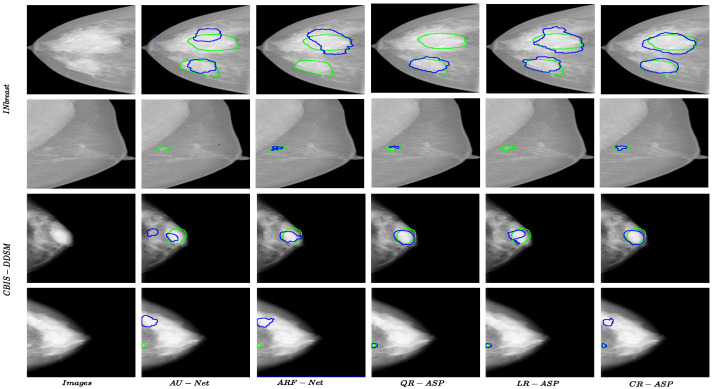
Results for R-ASP variants, AU-Net, and ARF-Net approaches for both datasets. The green color shows the border of ground truth and the blue color presents the prediction.

Regarding CBIS-DDSM, while the best results for all of the metrics were achieved by variations of R-ASP, QR-ASP was the best-performing variation, as shown in Table 3. This showed that while being generally effective, the degree of effectiveness was related to the appropriate grouping method, given the distribution of data. Figure 5 presents a few samples of the performance of different approaches for small and large size masses for both of the datasets. The best-performing R-ASP and D-ASP versions provided improvements over the baseline method for INbreast and CBIS-DDSM.

#### 4.2.2. Experimental Results for D-ASP

The results for D-ASP on INbreast and CBIS-DDSM are summarized in Table 4 and Table 5, respectively. D-ASP outperformed other methods using traditional pixel-level hybrid loss functions. Notably, the improvements over the baseline hybrid losses were as follows: DSC: +9.27%, ΔA: −12.77%, Sensitivity: +20.21%, Accuracy: +0.19%. Compared with R-ASP, D-ASP performed better in terms of DSC, ΔA, and sensitivity. For the CBIS-DDSM dataset, D-ASP also improved on the baseline method and ARF-Net (Table 5). However, the results were comparable to R-ASP. One observation was that D-ASP outperformed R-ASP in sensitivity on both datasets. For instance, when the DSCs were comparable, the sensitivity was considerably higher for D-ASP. The reason could be that the reduction in false positives was stronger in R-ASP, while in D-ASP, false negatives were more diminished. It should be noted that while both R-ASP and D-ASP shared the main idea of prioritizing the loss terms, they differed in the information that was used for the re-weighting signal and the losses used. In addition, for R-ASP, the re-weighting depended on the samples’ ratios and the interpretation of the ratios in a group of samples (training set). On the other hand, for D-ASP, the re-weighting signal depended on the information of the sample itself. Therefore, the difference in performance could be attributed to all of these factors.

**Figure 6:**
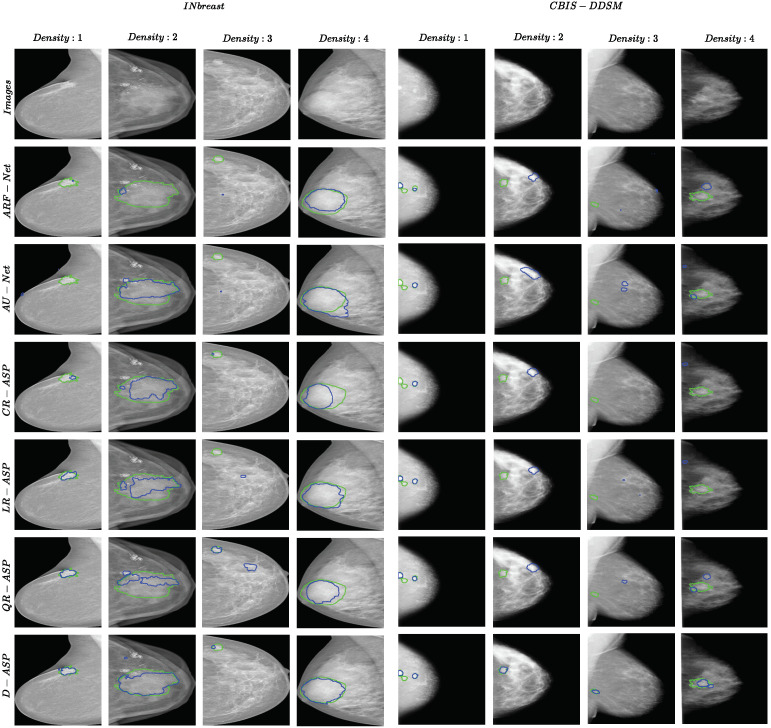
Examples of segmentation results for ARF-Net, AU-Net, R-ASP, and D-ASP for both datasets. One sample from each ACR density category is included. The green color shows the border of ground truth and the blue color presents the prediction.

Figure 6 presents one example from each ACR category for both datasets. One observation in all the samples was that the borders of D-ASP’s segmentations matched the borders of the true mass better compared with all of the other methods and R-ASP. This could be because incorporating the region-level losses enabled the loss to include the context in the calculation and eliminated false predictions.

## 5. Discussion

While ASP losses enhanced the performance of the baseline method, there were a few challenges that might have limited their impact. For instance, R-ASP variations depended on the grouping strategy; therefore, the performance could be improved by selecting the appropriate grouping strategies. Regarding D-ASP, one observation is that the ACR density category in some samples might not have been aligned with the expectation of visual differences, which was important for region-level losses. Hence, an inaccurate assessment of the density or any mismatch might have limited the performance of D-ASP. For both the ratio and density-based ASPs, the accuracy of the segmentation mask was important. This was a limitation regarding the CBIS-DDSM dataset, in which the segmentation masks were less accurate compared with INbreast. Regarding the computational time, the proposed method introduced a trivial additional time to the training, as the ratio and density had already been calculated for the training data and only re-weighting was performed during the training time.

## 6. Conclusions

In this paper, we propose using additional information in each sample as a re-weighting signal for prioritizing one of the loss types, which is more useful for the sample in an adaptive manner. We introduce two variations for the proposed loss. In one variation, called R-ASP, the ratio of the size of the mass to the image size is used for prioritizing dice loss over BCE, and vice versa. This version is based on the assumption that dice loss could be more useful for handling pixel class imbalance, and BCE could be more useful for samples with less severe pixel class imbalance. In the second variation, the density of each sample is utilized as a re-weighting signal in a hybrid loss with pixel-level and region-level loss terms. The rationale behind this variation is the fact that segmentation in samples with a higher ACR density is more challenging; therefore, region-level losses that utilize the information in the neighboring pixels while calculating the loss value for a central pixel can be beneficial for samples with a high ACR density. Both R-ASP and D-ASP are proposed for hybrid losses. In order to use the sample-level adaptation of ASP, we also introduce a variation in focal loss that uses the sample-level information (ratio and density) for the selection of the focusing parameter.

According to the results on both datasets for APS losses, the idea of using sample-level prioritizing loss in addition to including the region-level loss is a promising approach for improvement of the training, and it results in a boost in performance. Using ASP for focal loss also results in a promising performance enhancement. Both R-ASP and D-ASP loss could be generalized for other tasks in medical imaging.

## Figures and Tables

**Table 1:** Results for experiments for SR-ASP, SD-ASP, and VR-ASP on INbreast. In each subsection of the table, the best results for each metric have been shown in bold.

Losses	DSC	ΔA	Sensitivity	Accuracy
Focal	54.67	46.8	42.49	98.19
**SR-ASP**	**68.83**	26.63	**62.90**	**98.55**
SD-ASP	66.79	**16.41**	62.68	98.47
Hybrid	65.32	23.68	57.95	**98.46**
VR-ASP	**66.94**	**20.81**	**64.77**	98.43

**Table 2:** Comparison of R-ASP with state-of-the-art approaches for INbreast. The best results for each metric have been shown in bold.

Method	DSC	ΔA↓	Sensitivity	Accuracy
ARF-Net	70.05	30.37	59.59	98.71
AU-Net	65.32	23.68	57.95	98.46
QR-ASP	68.03	25.04	63.12	98.54
LR-ASP	71.92	22.31	64.56	98.71
**CR-ASP**	**74.18**	**19.28**	**67.21**	**98.78**

**Table 3:** Comparison of R-ASP with state-of-the-art approaches for CBIS-DDSM. The best results for each metric have been shown in bold.

Method	DSC	ΔA↓	Sensitivity	Accuracy
ARF-Net	48.82	11.47	47.27	99.43
AU-Net	49.05	09.94	51.49	99.38
**QR-ASP**	**51.48**	**02.05**	**52.00**	99.43
LR-ASP	51.33	23.17	45.38	**99.50**
CR-ASP	51.04	04.47	49.90	99.45

**Table 4:** Results for D-ASP, R-ASP and state-of-the-art approaches for INbreast. The best results for each metric have been shown in bold.

Method	DSC	ΔA↓	Sensitivity	Accuracy
ARF-Net	70.05	30.37	59.59	98.71
AU-Net (baseline)	65.32	23.68	57.95	98.46
QR-ASP	68.03	25.04	63.12	98.54
LR-ASP	71.92	22.31	64.56	98.71
CR-ASP	74.18	19.28	67.21	**98.78**
**D-ASP**	**74.59**	**10.91**	**78.16**	98.65

**Table 5:** Results for D-ASP, R-ASP and state-of-the-art approaches for CBIS-DDSM. The best results for each metric have been shown in bold.

Method	DSC	ΔA↓	Sensitivity	Accuracy
ARF-Net	48.82	11.47	47.27	99.43
AU-Net (baseline)	49.05	09.94	51.49	99.38
QR-ASP	**51.48**	**02.05**	52.00	99.43
LR-ASP	51.33	23.17	45.38	**99.50**
CR-ASP	51.04	04.47	49.90	99.45
**D-ASP**	50.64	05.96	**52.15**	99.41

## Data Availability

INbreat and CBIS-DDSM datasets are publicly available at https://www.kaggle.com/datasets/tommyngx/inbreast2012 (accessed on 1 January 2023) and https://wiki.cancerimagingarchive.net/pages/viewpage.action?pageId=22516629 (accessed on 1 January 2023), respectively.
